# Hyperalgesia and Central Sensitization in Subjects With Chronic Orofacial Pain: Analysis of Pain Thresholds and EEG Biomarkers

**DOI:** 10.3389/fnins.2020.552650

**Published:** 2020-11-12

**Authors:** Andrea Baroni, Giacomo Severini, Sofia Straudi, Sergio Buja, Silvia Borsato, Nino Basaglia

**Affiliations:** ^1^Translational Neurosciences and Neurotechnologies, Ferrara University, Ferrara, Italy; ^2^Department of Neuroscience and Rehabilitation, University Hospital of Ferrara, Ferrara, Italy; ^3^School of Electrical and Electronic Engineering, University College Dublin, Dublin, Ireland; ^4^Centre for Biomedical Engineering, University College Dublin, Dublin, Ireland

**Keywords:** chronic pain, orofacial pain, central sensitization, pain threshold, EEG

## Abstract

**Introduction:** The presence of a temporomandibular disorder is one of the most frequent causes of orofacial pain (OFP). When pain continues beyond tissue healing time, it becomes chronic and may be caused, among other factors, by the sensitization of higher-order neurons. The aim of this study is to describe psychological characteristics of patients with chronic OFP, their peripheral pain threshold, and electroencephalography (EEG) recording, looking for possible signs of central sensitization (CS).

**Materials and methods:** Twenty-four subjects with chronic OFP caused by temporomandibular disorder were evaluated using the Research Diagnostic Criteria for Temporomandibular Disorders Axis I and Axis II. Pain intensity, catastrophizing, and presence of CS were assessed through self-reported questionnaires. Pressure pain threshold (PPT) was recorded in facial and peripheral sites; EEG activity was recorded during open and closed eyes resting state and also during the pain threshold assessment. Pain thresholds and EEG recordings were compared with a cohort of pain-free age- and sex-matched healthy subjects.

**Results:** Patients with chronic OFP showed a significant reduction in their pain threshold compared to healthy subjects in all sites assessed. Greater reduction in pain threshold was recorded in patients with more severe psychological symptoms. Decreased alpha and increased gamma activity was recorded in central and frontal regions of all subjects, although no significant differences were observed between groups.

**Discussion:** A general reduction in PPT was recorded in people who suffer from chronic OFP. This result may be explained by sensitization of the central nervous system due to chronic pain conditions. Abnormal EEG activity was recorded during painful stimulation compared to the relaxed condition in both chronic OFP subjects and healthy controls.

## Introduction

Chronic pain is defined as pain that lasts for more than 3 months beyond the normal healing time (Treede et al., 2015). Chronic pain impacts working life, somatic, emotional and social well-being, and quality of life of the affected individuals and is recognized as a major health care problem in Europe (Breivik et al., 2006). Involvement of cerebral circuits in chronic pain development has been broadly documented (Apkarian et al., 2004; Kim et al., 2013; Ferdek et al., 2019). Chronic pain seems to be associated with pain related to central networks, and neuroplastic changes in these circuits may change perception of pain independent of peripheral neural activation (Camfferman et al., 2017). The thalamus appears to play a key role in several chronic pain conditions, and its connection with cerebral cortex seems imputable to maintenance of pain (Llinás et al., 1999; Stern et al., 2006). Many studies have tried to identify an electroencephalography (EEG) pattern related to pain development and maintenance beyond physiological tissue healing time (Prichep et al., 2011; Jensen et al., 2013; Pinheiro et al., 2016). Despite the lack of certainty around cortical markers of chronic pain, a reduction in alpha activity in frontal lobes and increased theta activity in the posterior parietal cortex have been recorded in subjects who experience chronic pain in various conditions (Sarnthein et al., 2006; Sarnthein and Jeanmonod, 2008; Jensen et al., 2013; Camfferman et al., 2017). Recently, the International Association for the Study of Pain (IASP) distinguished between “chronic primary pain” and “chronic secondary pain.” In the first category, chronic pain is conceived as a disease in its own right; in the second, pain is a consequence of an underlying disease and may be initially conceived as a symptom (Treede et al., 2019). Orofacial pain (OFP) is usually classified as chronic secondary pain because, in most cases, it can be attributed to an underlying cause (Benoliel et al., 2019). Frequently, the pain starts from a problem with the temporomandibular joint (TMJ), outlasts the initiating event, and becomes the leading cause for ongoing treatment (Benoliel et al., 2019). Patients, following temporomandibular disease (TMD) resolution, no longer exhibit peripheral tissue damage but continue to feel pain, suggesting an abnormal functioning of the somatosensory system (Sarlani and Greenspan, 2005). This process may be due to an induced sensitization of higher-order neurons, a phenomenon well described by the central sensitization (CS) process (Campi et al., 2017). According to the IASP definition, CS is characterized by an increased responsiveness of nociceptive neurons in the central nervous system (CNS) to their normal or subthreshold afferent input (Loeser and Treede, 2008). With the introduction of the CS concept, pain starts to reflect a functional state of circuits in the CNS instead of being exclusively peripherally driven (Woolf, 2011). Injury or inflammation in peripheral tissue can alter the properties of somatic sensory pathways. This induced peripheral sensitization could trigger CS, leading to pathological pain states (Harte et al., 2018). Evidence for CS has been described in patients with TMD by Dworkin (1995), who found no correlation between physical signs of jaw dysfunction and levels of pain in a 3-year follow-up study. Quantitative sensory testing, such as pressure pain threshold (PPT), can be used to document the patient’s somatosensory profile (Svensson et al., 2011). A generalized state of pain sensitivity can justify low PPT, linked to altered sensory processing, dysregulated endocrine function, hyperinflammatory states, or psychological processes (Lautenbacher et al., 1994). In a large prospective study, the OPPERA (Orofacial Pain: Prospective Evaluation and Risk Assessment) study, Slade et al. (2014) observed that PPT fluctuated in synchrony with the course of painful TMD. Further, a reduction of PPT in sites related to the TMJ has been identified as sign of peripheral sensitization (Campi et al., 2017). In case of sensitization due to supraspinal pathways, the local threshold is further reduced at the local site, but it is also reduced in more distant body sites not related to TMD. The comparison of a TMD cohort with a healthy and pain-free sample may be the only way to evaluate the degree of localized and spreading sensitization (Arendt-Nielsen et al., 2018). We can assume that changes in EEG activity and signs of sensitization can be recorded in people who suffer from long-lasting pain due to TMD. The objective of this study is therefore to describe features of chronic OFP through the analysis of patients’ psychological profile, peripheral pain threshold, and EEG recordings, looking for possible signs of CS.

## Materials and Methods

This cross-sectional observational study describes factors related to chronic OFP and characteristics of patients in a cohort of 24 subjects with OFP due to TMD. This study has been reviewed by the Ferrara University Hospital Ethics Committees. All the procedures described have been carried out in accordance with the Code of Ethics of the World Medical Association (Declaration of Helsinki) for experiments involving humans. Written informed consent was obtained before all procedures. The study meets the STROBE Guidelines for observational studies (von Elm et al., 2014).

Patients who underwent rehabilitation for TMD at Ferrara Rehabilitation Hospital between January 2018 and January 2019 were assessed for eligibility. Age, sex, occupation, side and duration of TMD, past treatment for the TMJ, comorbidities, and medications were recorded. All subjects with a Numeric Pain Rating Scale (NPRS) of less than 3 in the 2 weeks prior to assessment or who took pain relief medication were excluded from the study (Jensen et al., 2013). The other exclusion criteria were impaired cognitive functioning (score < 24 on the Mini-Mental Status Examination), neurological or psychiatric disorders, or pregnancy.

A medical doctor with an expertise in temporomandibular rehabilitation evaluated all subjects included in the study, following the Research Diagnostic Criteria for Temporomandibular Disorders (RDC/TMD) Axis I (Schiffman et al., 2014).

The RDC/TMD Axis II was used to assess psychological distress and pain-related disability (Schiffman et al., 2014). For the purpose of this analysis, depression, anxiety, and non-specific physical symptoms (NSPS) were treated as dichotomous variables, and patients were classified as minimal/mild if their total score was lower than 10; patients with a higher score were classified as moderate/severe (Campi et al., 2017). All subjects included were evaluated using a self-reported questionnaires for subjective description of pain and PPT for objective assessment of pain perception (Dworkin et al., 2005). Neural activity linked to pain sensation was recorded using EEG. PPT and EEG were also evaluated in a sample of age- and sex-matched healthy controls.

### Self-Reported Questionnaire

Catastrophizing has been defined as “an exaggerated negative orientation toward actual or anticipated pain experiences” and reflects a tendency to misinterpret or exaggerate apparently threatening situations (Sullivan et al., 1995). The Pain Catastrophizing Scale (PCS) was used to assess the tendency to magnify the threat value of pain stimulus and to feel helpless in the context of pain (Quartana et al., 2009). A PCS score ≥30 was used to detect the presence of catastrophizing (Sullivan et al., 1995).

Central sensitization was assessed using the Italian version of the Central Sensitization Inventory (CSI-I) (Chiarotto et al., 2018). A CSI score ≥40 has been suggested as the cutoff score to determine if patients display CS (Neblett et al., 2013, 2015; Nijs et al., 2014).

### Pressure Pain Threshold

**FIGURE 1:**
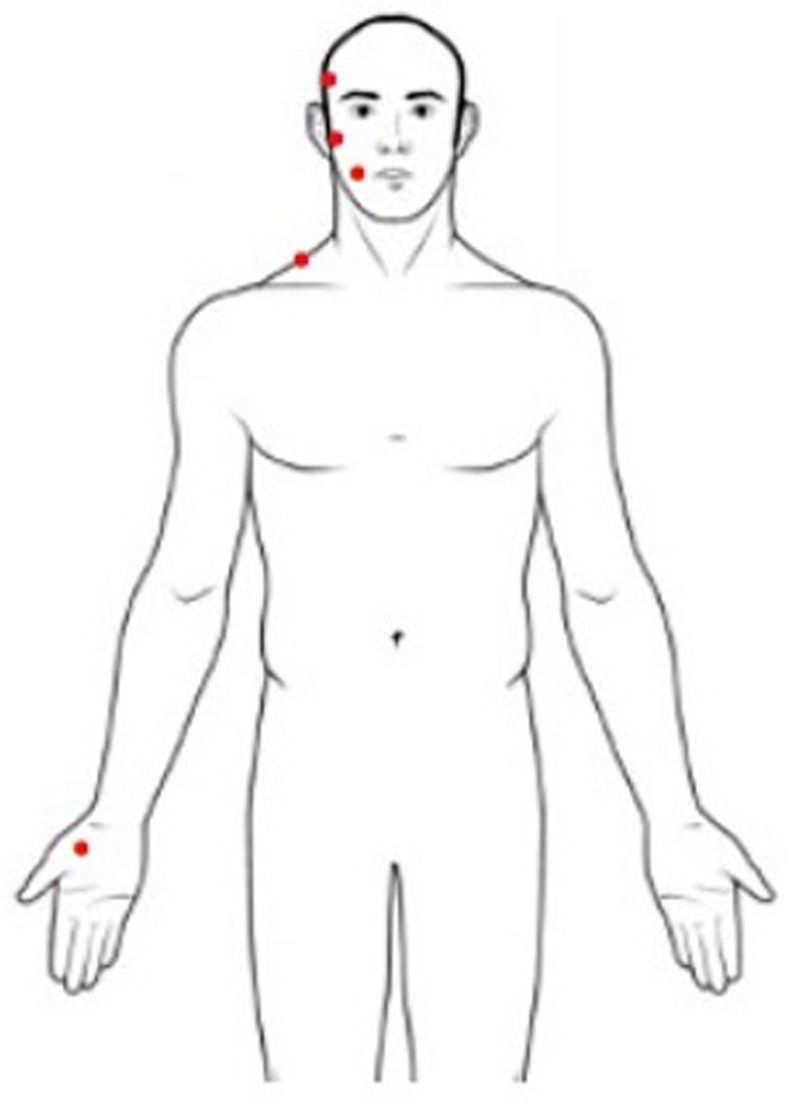
Sites for pressure pain threshold assessment (right body sites for illustrative purposes only).

Pressure pain threshold is defined as the minimum pressure applied to anatomical regions that can induce pain (Fischer, 1987). PPT measurement was performed with a handled digital dynamometer (Commander Algometer, JTECH Medical, United States), consisting of a device with a 1-cm^2^ flat circular tip used to apply pressure on subjects’ skin. A researcher was trained to apply increasing pressure of approximately 1 lb/cm^2^/s perpendicular to the skin using the dynamometer, following a protocol well described in literature (Campi et al., 2017). The stimulus intensity increased from zero, and the subject was instructed to stop the stimulation at the first perception of pain by pushing a button. At that moment, the pressure was removed, and the value of pressure applied was recorded. The sites of the stimulation were the muscle belly of the temporal and masseter muscles, the surface of the mandibular condyle, the middle part of the upper trapezius, and the center of the thenar eminence (Figure 1). During examinations subjects were in a comfortable sitting position with muscles relaxed. The researcher stabilized the subject’s head gently applying manual resistance contralateral to the point of pressure application. This procedure was repeated three times for every site, on both sides, with an interstimulus interval of 30 s (Nie et al., 2009). The PPT value of the painful side was used for the analysis. When symptoms were present bilaterally, the value of the more affected side was used. This side was matched in measuring PPT in healthy subjects.

### EEG Recording

Electroencephalography assessment was performed using an electrode montage of 32 Ag/AgCl pellet pin electrodes (Easy Cap GmbH, Herrsching, Germany) placed according to the 10–20 International System on a Fast’n Easy cap. A BrainAmp amplifier (Brain Products, Munich, Germany) was used to record EEG activity. All scalp electrodes were referenced to nasion and grounded at AFz during recordings. Horizontal and vertical eye movements were detected, respectively, with electrodes placed at the left and right outer canthi at Fp1 and below the eye at the non-painful side. The impedance of all the electrodes was kept below 10 kΩ. The EEG signals were recorded with a 1,000-Hz sampling rate with a low cutoff frequency of 0.1 Hz and a high cut-off of 1,000 Hz.

Electroencephalography data were recorded during a 5-min resting state task with open eyes and a 5-min resting state task with closed eyes. Participants were instructed to stay relaxed and keep their eyes fixed on a cross in front of them during open-eyes recording. EEG was also recorded during the PPT assessment at the thenar eminence following the aforementioned protocol.

### EEG Preprocessing

The EEG data were preprocessed in MATLAB, using the EEGlab toolbox (Delorme and Makeig, 2004). A notch filter centered around 50 Hz was applied in post-processing for eliminating the power noise. Then, data were re-referenced to the average reference. Eye movement artifacts were removed by means of an independent component analysis (ICA) procedure. ICA was used to determine the independent components. A visual analysis was used to discard components that were characterized by high-amplitude fluctuations and were mostly located at or close to the eye electrodes.

### EEG Spectral Analysis

The spectral power in the different EEG bands (delta 1–4 Hz, theta 4–7 Hz, alpha 7–13 Hz, beta 13–30 Hz, gamma 30–60 Hz) was calculated, during both resting state tasks, in the middle minute of the 5 min of each recording. The power spectral density (PSD) was calculated using Welch’s method, using 1-s windows and 80% of overlap over successive windows (Welch, 1967). The PSDs of all subjects during each trial were then transformed into *z*-scores to improve comparability of PSD values across subjects and conditions. For the pain stimulus trials, the PSD was calculated from the 3-s window before reaching the sensory threshold. The *z*-score of the PSD was calculated for each electrode of each subject during each condition by subtracting by each PSD spectrum its mean and dividing by its standard deviation. For the statistical analyses, in order to minimize the number of comparisons, we calculated the average PSD in the different bands for clusters of electrodes centered around locations F3, C3, P3, and their homologous on the right side of the scalp. For each location, the PSD in each band was calculated as the average PSD in the band among the central and directly adjacent electrodes. To visually assess for relative differences in EEG activity due to the pain, the *z*-score PSD calculated during the pain stimulus trials was expressed as a percentage of the average PSD calculated from the resting state trials with the eyes open of all subjects. This choice for normalization was dictated by the fact that subjects had their eyes open during the pain stimulus trials. These data were then plotted for both groups for visual comparison.

### Statistical Analysis

Descriptive statistics were used for characterizing the sample. Continuous variables are reported as means and standard deviations, non-continuous variables as counts and percentages. Differences in PPT between patients with OFP and healthy subjects were assessed using the Wilcoxon rank-sum test due to non-normal data distribution. Patients with OFP were also divided according to intensity of pain, presence of psychological disorders, catastrophizing, and CS, and differences between groups were analyzed. Spearman rank correlation coefficient was used to measure strength and direction of association between psychological scores and self-reported questionnaires.

This statistical analysis was performed using STATA 13.1 software with significance set at ?? < 0.05. Statistical analysis was also performed on the clustered EEG data. In this analysis, we compared the *z*-score PSD of the OFP and the healthy subjects in the eyes open and pain stimulus conditions for each band and electrode cluster. This analysis was based on a two-way analysis of variance (ANOVA) test. The significance level was set to 0.05. All the statistical analysis on the EEG data was performed in MATLAB using custom-made scripts.

## Results

**FIGURE 2:**
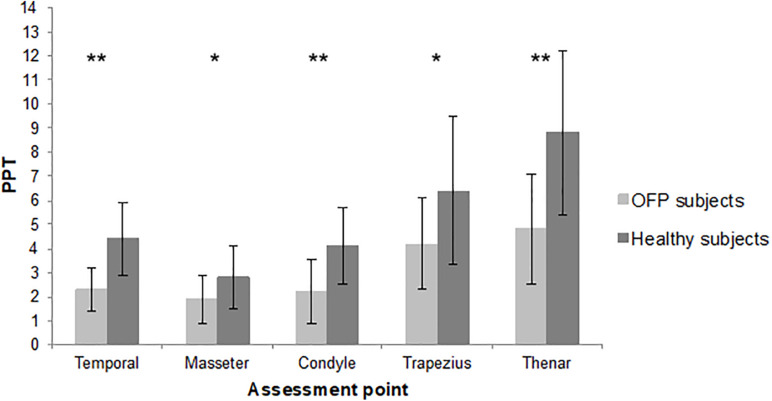
Mean pressure pain threshold of the two samples. PPT, pressure pain threshold; lb, libre; OFP, orofacial pain; *p < 0.01; **p < 0.001.

The sample consisted of 19 women and 5 men. The mean age was 49.8 years, with a minimum of 23 and a maximum of 77 years. Detailed demographic and clinical features of the sample are summarized in Table 1. Most of the sample was classified as myofascial pain with spreading following the Axis 1 of DC/TMD. The mean pain intensity during the 24 h before at the NPRS was 6.42 (1.72 SD), with a minimum of 3 and a maximum of 9. Twenty-four age- and sex-matched healthy subjects were recruited. The assessment of PPT revealed a reduction in pain threshold in subjects with OFP in all the sites of assessment compared to healthy subjects. Differences between groups were statistically significant (Figure 2). Reduction in PPT in subjects with OFP compared to healthy subjects was observed even after removing people with fibromyalgia from the analysis (*p* < 0.05 for all the sites of assessment). Stratifying patients according to psychological assessment performed with RDC/TMD Axis II, we observed differences in PPT between groups of subjects with moderate or severe symptoms compared to those with low or mild; significant differences were recorded only for pain-related disability and depression (*p* = 0.045 and *p* = 0.023, respectively) (Table 2). No significant differences in pain threshold were identified in patients with CS signs. Positive correlations were found between CS and psychological disorders for every class of impairment ρ = 0.331 for depression, ρ = 0.575 for NSPS, ρ = 0.365 for catastrophizing), without reaching significant level.

**TABLE 1:** Descriptive data for the sample.

	Sample (*n* = 24) Mean (SD) or *n* (%)
Age (years)	49.8 (13.1)
Sex (*n*)	
Male/female	5 (21)/19 (79)
Occupation (*n*)	
Employed/unemployed	14 (58)/10 (42)
Principal comorbidities (*n*)	
Fibromyalgia	5 (21)
Hypertension	4 (17)
Enteric disease	3 (12)
Diabetes mellitus	1 (4)
Other rheumatic disease	1 (4)
None	10 (42)
Drug use (*n*)	
Non-steroidal anti-inflammatory drugs	5 (21)
Antidepressants	2 (8)
Muscle relaxants	2 (8)
Analgesics	3 (11)
None	12 (50)
Symptoms duration (months)	49.21 (68.59)
Symptoms frequencies (*n*)	
Continuous/episodic recurrent	7 (35)/17 (65)
Pain side (*n*)	
Right/left/bilateral	3 (10)/3 (15)/18 (75)
Previous treatment	
Physiotherapy	11 (45)
Arthrocentesis	10 (35)
Byte use	16 (65)
DC/TMD Axis I	
Myofascial pain	21 (90)
Myalgia	1 (5)
Arthralgia	2 (5)
DC/TMD Axis II	
Pain-related disability (*n*)	
Low	9 (37.5)
High	15 (62.5)
Depression (*n*)	
Minimal–mild	16 (66.7)
Moderate–severe	8 (33.3)
Anxiety (*n*)	
Minimal–mild	19 (79)
Moderate–severe	5 (21)
Non-specific physical symptoms (*n*)	
Minimal–mild	13 (54)
Moderate–high	11 (46)
NPRS	6.42 (1.72)
Catastrophizing (*n*)	
Not present	12 (50)
Present	12 (50)
Central sensitization (*n*)	
Subclinical–mild	12 (50)
Moderate–severe	12 (50)

**n*, number; SD, standard deviation; DC/TMD Axis I and Axis II, Diagnostic Criteria for Temporomandibular Disorders Axis I and Axis II; NPRS, Numeric Pain Rating Scale.*

**TABLE 2:** Pressure pain threshold for classes of impairment.

		Mean (SD)	*p*
DC/TMD Axis II			
Pain-related disability	Low (*n* = 9)	3.8 (1.3)	0.045*
	High (*n* = 15)	2.6 (1.1)	
Depression	Minimal–mild (*n* = 16)	3.5 (1.3)	0.023*
	Moderate–severe (*n* = 8)	2.3 (1.0)	
Anxiety	Minimal–mild (*n* = 19)	3.1 (1.3)	0.749
	Moderate–severe (*n* = 5)	2.9 (1.3)	
Non-specific physical symptoms	Minimal–mild (*n* = 13)	3.4 (1.4)	0.213
	Moderate–severe (*n* = 11)	2.7 (1.1)	
NPRS	Mild–moderate 3–6 (*n* = 12)	3.0 (1.3)	0.954
	Severe 7–10 (*n* = 12)	3.1 (1.3)	
Catastrophizing	Not present (*n* = 12)	3.2 (1.4)	0.427
	Present (*n* = 12)	2.8 (0.9)	
Central sensitization	Subclinical–mild (*n* = 12)	3.3 (1.5)	0.564
	Moderate–severe (*n* = 12)	2.9 (0.9)	

*n*, number; SD, standard deviation; DC/TMD Axis II, Diagnostic Criteria for Temporomandibular Disorders Axis II; NPRS, Numeric Pain Rating Scale. *p < 0.05.

### EEG Results

**FIGURE 3:**
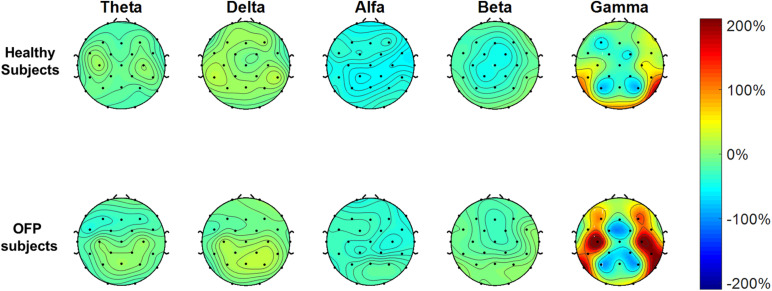
Electrode-level maps of the percentage changes in the *z-*values of the power spectral density in the different bands between the open eyes resting task and the pain stimulus task. The top row represents the healthy controls; the bottom row, the orofacial pain (OFP) patients.

The average PSD *z*-score values for OFP and healthy individuals for both conditions are presented in Table 3. We did not notice specific qualitative trends in the PSD values between OFP and healthy controls, which translated in the absence of statistically significant differences in the ANOVA in the PSD calculated from all electrode clusters in all frequency bands among the two groups (Table 4). However, we observed a marked decrease in PSD values for both groups between the two conditions in the alpha and beta bands. This observed decrease in PSD translated in statistically significant differences in the ANOVA between the two conditions for all clusters in the alpha band and for clusters F3 and C3 in the beta band. In the gamma band, we observed a general qualitative trend of increased PSD values in both groups in most clusters. This trend translated in statistically significant differences in the ANOVA for all clusters (with the exception of P4) in the gamma band. The interaction analysis (Table 4) suggests that the differences observed in the gamma band are group-specific. We then analyzed the group-specific relative changes in PSD values due to the pain stimulus. This was done by expressing the *z*-score PSD values extracted during the PS trial and expressed as percentage changes with respect to the same values extracted during the EO trial. We observed a qualitative increase in the relative PSD values (with respect to the eyes open trial) in the gamma band in the controls that were localized mostly in the occipital region. In the patients, differently than the controls, increased values of PSD in the gamma band were instead observed in the central and frontal regions (C3/C4 F3/F4 electrodes) (Figure 3).

**TABLE 3:** PSD values, expressed as mean (standard deviation) of the *z*-scores, for the different clusters, across all frequency bands, for the orofacial pain (OFP) patients and the healthy controls (HC) for the eyes open (top table) and pain stimulus (bottom table) conditions.

Eyes Open										
	Theta		Delta		Alfa		Beta		Gamma	
Cluster	OFP	HC	OFP	HC	OFP	HC	OFP	HC	OFP	HC
*F3*	0.67 (0.73)	0.75 (0.72)	1 (0.74)	1.2 (0.62)	0.78 (0.94)	0.78 (0.99)	0.15 (0.35)	0.21 (0.37)	−0.46 (0.11)	−0.5 (0.12)
*F4*	0.68 (0.66)	0.77 (0.58)	1.1 (0.64)	1.2 (0.63)	0.83 (0.91)	0.78 (0.89)	0.1 (0.35)	0.25 (0.38)	−0.42 (0.099)	−0.51 (0.13)
*C3*	0.52 (0.67)	0.66 (0.6)	1 (0.75)	1.2 (0.54)	1.1 (0.93)	1.1 (0.97)	0.18 (0.42)	0.22 (0.3)	−0.45 (0.095)	−0.47 (0.088)
*C4*	0.54 (0.65)	0.62 (0.5)	1.1 (0.7)	1.2 (0.6)	1.1 (0.93)	1.1 (0.95)	0.14 (0.45)	0.24 (0.32)	−0.41 (0.16)	−0.49 (0.098)
*P3*	0.67 (0.53)	0.8 (0.45)	1.1 (0.62)	1.3 (0.44)	1.5 (1)	1.3 (0.83)	0.16 (0.41)	0.16 (0.23)	−0.45 (0.077)	−0.47 (0.054)
*P4*	0.67 (0.55)	0.76 (0.42)	1.1 (0.69)	1.2 (0.48)	1.5 (1)	1.3 (0.85)	0.13 (0.42)	0.16 (0.21)	−0.43 (0.1)	−0.47 (0.072)
**Pain stimulus**										
*F3*	0.3 (0.52)	0.58 (0.51)	0.86 (0.68)	1.2 (0.55)	0.38 (0.69)	0.27 (0.5)	0.049 (0.3)	0.016 (0.24)	−0.4 (0.11)	−0.39 (0.11)
*F4*	0.43 (0.53)	0.55 (0.45)	1.1 (0.71)	1.1 (0.49)	0.31 (0.57)	0.19 (0.43)	0.067 (0.38)	0.03 (0.28)	−0.41 (0.13)	−0.39 (0.14)
*C3*	0.4 (0.37)	0.54 (0.5)	1.1 (0.57)	1.2 (0.62)	0.57 (0.8)	0.34 (0.5)	0.092 (0.33)	-0.0018 (0.17)	−0.41 (0.11)	−0.36 (0.12)
*C4*	0.44 (0.48)	0.47 (0.5)	1.2 (0.6)	1.1 (0.6)	0.45 (0.56)	0.25 (0.38)	0.086 (0.35)	0.036 (0.21)	−0.41 (0.12)	−0.36 (0.14)
*P3*	0.57 (0.36)	0.59 (0.5)	1.1 (0.43)	1.3 (0.52)	0.82 (0.77)	0.51 (0.47)	0.12 (0.33)	0.035 (0.19)	−0.44 (0.088)	−0.39 (0.085)
*P4*	0.57 (0.38)	0.52 (0.54)	1.3 (0.57)	1.2 (0.59)	0.86 (0.79)	0.55 (0.59)	0.084 (0.3)	0.062 (0.18)	−0.43 (0.074)	−0.4 (0.087)

**TABLE 4:** Results of the ANOVA two-way analysis performed between groups [orofacial pain (OFP) and healthy controls (HC)], conditions [eyes open (EO) and pain stimulation (PS)], and the interaction between group and condition, expressed as *p-*value and the relative *F* statistic in parenthesis.

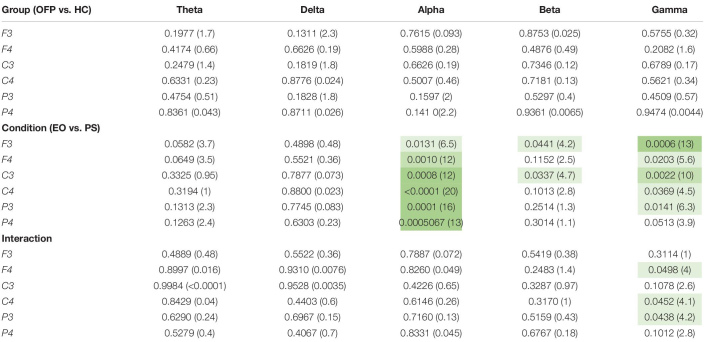

*The three different shades of green represent three *p*-value threshold, specifically *p* < 0.05 (lightest shade), *p* < 0.01 and *p* < 0.001 (darkest shade).*

## Discussion

In this observational study, we tried to describe features and clinical signs of people with chronic OFP due to TMD by comparing them with healthy controls. Our main finding revealed that people who suffer from this debilitating condition present a generalized reduction in PPT. This reduction in pain threshold was observed not only in facial sites but also in areas not involved by pathology, such as the upper trapezius and the thenar eminence. The phenomenon we observed may be due to CS, an increased responsiveness of nociceptive neurons to subthreshold input (Loeser and Treede, 2008). Fillingim et al. (2018) in their longitudinal study found that individuals who transitioned from being TMD-free to a TMD state tended to show reduction in PPT limited to the orofacial region and not to other body sites. The discrepancy between these and our results may be explained by the difference in time elapsed between OFP onset and PPT assessment in the two studies.

Fillingim et al.’s (2018) research did, in fact, involve a long-term follow-up, in which years separated original and follow-up assessments.

Again from the OPPERA study, Greenspan et al. (2011) found that people with chronic TMD were more pain-sensitive than controls to many mechanical and thermal stimuli, with particular sensitivity to pressure stimulation, applied to symptomatic and asymptomatic body sites.

In our study, PPT was assessed in patients with OFP from a median time of 33 months. Chronic pain, critical in development of CS, has to last for more than 3 months to be defined as such (Treede et al., 2015). Pain lasting for a shorter time may not contribute to hyperexcitability of the CNS, one of the main features of sensitization process (den Boer et al., 2019). In our study, we included patients with fibromyalgia, and this may represent a confounding factor in PPT assessment (Maquet et al., 2004). However, the analysis performed on the sample after exclusion of fibromyalgia patients showed no differences compared with the whole sample. Stratification of people with OFP based on psychological disorder severity revealed that subjects with moderate or severe depression and high level of pain-related disability showed generalized reduction in PPT. A large systematic review on pain sensitivity and depression found uncertain results about mechanisms underlying their relationship (Thompson et al., 2016). However depression and pain sensitivity frequently occur together (Von Knorring et al., 1983; Von Korff et al., 1988; Bair et al., 2003; Lépine and Briley, 2004; Agüera-Ortiz et al., 2011), probably due to dysfunction at the level of the serotonergic and noradrenergic neurons that affects not only psychological and somatic symptoms of depression but also physical painful symptoms (Stahl and Briley, 2004). Another possible explanation of the aforementioned results is that depressed people react negatively to painful stimulation with stronger emotional involvement. A reduction in PPT as sign of CS may explain the link between sensitization of the CNS and emotional comorbidities. Smart et al. (2012) in their study on patients with low back pain reported significantly greater levels of pain-related disability, depression, and anxiety in people with signs of CS compared to those with nociceptive or neuropathic pain. Strong relationship between CS and psychological symptoms is confirmed by our analysis. What needs to be clarified is the causal link between them, establishing if psychological disorders are involved in causing sensitization or whether they are a consequence of a sensitized system.

To our knowledge, the current study is the first investigating EEG PSD during PPT assessment in people with OFP vs. healthy control subjects. In this study, we found no differences between patients and control subjects during resting and pain stimulus trials. Our results about the reduction in the EEG alpha power during pain stimulation, which we observed in both cohorts, had already been observed in literature, without distinction between phasic or tonic pain (Chang et al., 2002; Peng et al., 2015). We observed also an increase in gamma band activity across all the electrodes between the two conditions. This translated, in the OFP patients, in a qualitative relative (with respect to the resting condition) increase in the central and prefrontal activity in the gamma band during peripheral stimulation just before stimulus was perceived as painful. Other studies investigating resting state EEG in people with chronic pain described significant overactivation of regions involved in the pain network. Prichep et al. (2018) recorded overactivity in insula areas, parietal lobule, thalamus, and the dorsolateral prefrontal cortex; significant differences between normal and pain patients were found in mid and posterior cingulate. Generalized overactivity was described in all areas belonging to the “pain matrix.” Our findings of an increase in gamma activity in the prefrontal areas may further support the model proposed by Baliki and Apkarian (2015) on dissociation in processing of longer lasting pain and nociceptive information. The authors described a dissociation of prefrontal component of the default mode network (DMN) in different types of chronic pain (Baliki and Apkarian, 2015). In fMRI studies, the DMN was described as one of the three brain systems that, with their dynamic interactions, are involved in spontaneous attentional fluctuations toward and away from pain (Kucyi and Davis, 2015). The DMN is activated when subject attention is not engaged by sensations from the external world (Andrews-Hanna et al., 2014). In opposition to the DMN, a system known as the salience network (SN) works to track how external stimuli capture attention (Downar et al., 2000, 2001, 2002, 2003; Mouraux et al., 2011; Uddin, 2015). Prefrontal areas, in particular dorsolateral prefrontal cortex, are part of the SN (Seeley et al., 2007; Kucyi et al., 2012). Although in our study we did not observe statistically significant differences between patients and controls, an overactivity of the prefrontal cortex recorded in patients with OFP due to TMD may be representative of an exaggerated engagement of SN in people with long-lasting pain and a general tendency to focus attention on external stimuli that could generate pain. Similar results of increased prefrontal gamma activity were reported in chronic back pain patients (May et al., 2019) and patients with postherpetic neuralgia and fibromyalgia (Lim et al., 2016; Zhou et al., 2018). The association between gamma oscillations and involuntary attentional effects of pain has been well described in literature (Hauck et al., 2007; Tiemann et al., 2010; Schulz et al., 2015; Hansen et al., 2017) and has great relevance in cortical networks for behavioral and cognitive phenomena (Uhlhaas et al., 2009).

Increased activity of the primary motor cortex (M1) area in people with chronic pain has been previously described in literature in various musculoskeletal conditions (Di Pietro et al., 2013; Schabrun et al., 2015, 2017; Te et al., 2017). A recent systematic review found inconclusive results with regard to abnormal M1 activation in pain conditions due to the heterogeneity of studies and assessment tools (Chang et al., 2018). Our results seem to underlie abnormal brain activity recorded by C3/C4 electrodes just before the peripheral stimulus became painful. Increased gamma activity may indicate increased muscle activity during pain, which contaminates EEG signal during pain stimulation. However, we did not record muscular activity during pain threshold assessment (Whitham et al., 2007; Dowman et al., 2008). Furthermore, muscular activation would also be highlighted by altered EEG signal during the recording.

Movement dysfunction such as unnecessary protective behavior may justify our findings, when patients received a stimulus perceived as threatening. The primary motor cortex has already been target of brain stimulation treatment, with a positive impact on pain relief (Fregni et al., 2006; Straudi et al., 2018). Abnormal function of motor and prefrontal cortex during stimulus perception may be due to neuroplastic changes that occur in the human brain subjected to long-lasting pain. Neurophysiological adaptations occur and seem to persist over peripheral tissue healing time in presence of emotional and behavioral aspects of pain that cause maladaptive changes in areas not normally involved in pain perception (Mansour et al., 2014). Structural as well as functional changes have been described in frontal and motor areas of patients with chronic pain due to coxarthrosis (Rodriguez-Raecke et al., 2013).

Interpretation of our findings is subject to several limitations. First, the small sample size does not allow us to confirm our results on PPT and EEG recordings. Even though CS may be hypothesized looking at our results, we cannot draw any definitive conclusion on the mechanism underlying sensitization of CNS. Second, interpretation of our results must consider the inclusion in our sample of fibromyalgia patients whose sensitivity to pain may influence their PPT.

## Conclusion

In a convenience sample of patients with OFP due to TMD we observed generalized reduction in PPT compared to age- and sex-matched healthy controls, not limited to facial sites. Generalized decrease of pain threshold seems to be linked to the severity of psychological symptoms such as depression and perceived health-related disability. Abnormal EEG activity was recorded during painful stimulation of non-painful sites of patients with OFP due to TMD. This observational study tried to identify potential signs of CS through the analysis of patients’ sensory and psychological profiles and brain activity. Our results can open doors to new strategies for the assessment and treatment of patients with CS due to chronic pain conditions.

## Data Availability Statement

The raw data supporting the conclusions of this article will be made available by the authors, without undue reservation.

## Ethics Statement

The studies involving human participants were reviewed and approved by the Ferrara University Hospital Ethics Committees. The patients/participants provided their written informed consent to participate in this study.

## Author Contributions

AB, SS, SBu, and NB conceived the study and participated in its design. AB and SBo performed the instrumented and clinical data collections. AB and GS analyzed the data. AB, GS and SS interpreted the results, and drafted and revised the manuscript. All authors approved the submitted version.

## Conflict of Interest

The authors declare that the research was conducted in the absence of any commercial or financial relationships that could be construed as a potential conflict of interest.

## References

[B1] Agüera-Ortiz L. Failde I. Mico J. A. Cervilla J. López-Ibor J. J. (2011). Pain as a symptom of depression: prevalence and clinical correlates in patients attending psychiatric clinics. *J. Affect. Disord.* 130 106–112. 10.1016/j.jad.2010.10.022 21055826

[B2] Andrews-Hanna J. R. Smallwood J. Spreng R. N. (2014). The default network and self-generated thought: component processes, dynamic control, and clinical relevance. *Ann. N Y. Acad. Sci.* 1316 29–52. 10.1111/nyas.12360 24502540 PMC4039623

[B3] Apkarian A. V. Sosa Y. Sonty S. Levy R. M. Harden R. N. Parrish T. B. (2004). Chronic back pain is associated with decreased prefrontal and thalamic gray matter density. *J. Neurosci.* 24 10410–10415. 10.1523/JNEUROSCI.2541-04.2004 15548656 PMC6730296

[B4] Arendt-Nielsen L. Morlion B. Perrot S. Dahan A. Dickenson A. Kress H. G. (2018). Assessment and manifestation of central sensitisation across different chronic pain conditions. *Eur. J. Pain* 22 216–241. 10.1002/ejp.1140 29105941

[B5] Bair M. J. Robinson R. L. Katon W. Kroenke K. (2003). Depression and pain comorbidity: a literature review. *Arch. Intern. Med.* 163 2433–2445. 10.1001/archinte.163.20.2433 14609780

[B6] Baliki M. N. Apkarian A. V. (2015). Nociception. Pain, Negative Moods, and Behavior Selection. *Neuron* 87 474–491. 10.1016/j.neuron.2015.06.005 26247858 PMC4529956

[B7] Benoliel R. Svensson P. Evers S. Wang S.-J. Barke A. Korwisi B. (2019). The IASP classification of chronic pain for ICD-11: chronic secondary headache or orofacial pain. *Pain* 160 60–68. 10.1097/j.pain.0000000000001435 30586072

[B8] Breivik H. Collett B. Ventafridda V. Cohen R. Gallacher D. (2006). Survey of chronic pain in Europe: prevalence, impact on daily life, and treatment. *Eur. J. Pain* 10 287–333. 10.1016/j.ejpain.2005.06.009 16095934

[B9] Camfferman D. Moseley G. L. Gertz K. Pettet M. W. Jensen M. P. (2017). Waking EEG Cortical Markers of Chronic Pain and Sleepiness. *Pain Med.* 18 1921–1931. 10.1093/pm/pnw294 28087845 PMC6407607

[B10] Campi L. B. Jordani P. C. Tenan H. L. Camparis C. M. Gonçalves D. A. G. (2017). Painful temporomandibular disorders and central sensitization: implications for management-a pilot study. *Int. J. Oral. Maxillofac Surg.* 46 104–110. 10.1016/j.ijom.2016.07.005 27553896

[B11] Chang P.-F. Arendt-Nielsen L. Chen A. (2002). Dynamic changes and spatial correlation of EEG activities during cold pressor test in man. *Brain Res. Bull.* 57 667–675. 10.1016/S0361-9230(01)00763-8 11927371

[B12] Chang W.-J. O’Connell N. E. Beckenkamp P. R. Alhassani G. Liston M. B. Schabrun S. M. (2018). Altered Primary Motor Cortex Structure. Organization, and Function in Chronic Pain: A Systematic Review and Meta-Analysis. *J. Pain* 19 341–359. 10.1016/j.jpain.2017.10.007 29155209

[B13] Chiarotto A. Viti C. Sulli A. Cutolo M. Testa M. Piscitelli D. (2018). Cross-cultural adaptation and validity of the Italian version of the Central Sensitization Inventory. *Musculoskelet Sci. Pract.* 37 20–28. 10.1016/j.msksp.2018.06.005 29966856

[B14] Delorme A. Makeig S. (2004). EEGLAB: an open source toolbox for analysis of single-trial EEG dynamics including independent component analysis. *J. Neurosci. Methods* 134 9–21. 10.1016/j.jneumeth.2003.10.009 15102499

[B15] den Boer C. Dries L. Terluin B. van der Wouden J. C. Blankenstein A. H. van Wilgen C. P. (2019). Central sensitization in chronic pain and medically unexplained symptom research: A systematic review of definitions, operationalizations and measurement instruments. *J. Psychosom. Res.* 117 32–40. 10.1016/j.jpsychores.2018.12.010 30665594

[B16] Di Pietro F. McAuley J. H. Parkitny L. Lotze M. Wand B. M. Moseley G. L. (2013). Primary motor cortex function in complex regional pain syndrome: a systematic review and meta-analysis. *J. Pain* 14 1270–1288. 10.1016/j.jpain.2013.07.004 24035350

[B17] Dowman R. Rissacher D. Schuckers S. (2008). EEG indices of tonic pain-related activity in the somatosensory cortices. *Clin. Neurophysiol.* 119 1201–1212. 10.1016/j.clinph.2008.01.019 18337168 PMC2676940

[B18] Downar J. Crawley A. P. Mikulis D. J. Davis K. D. (2000). A multimodal cortical network for the detection of changes in the sensory environment. *Nat. Neurosci.* 3 277–283. 10.1038/72991 10700261

[B19] Downar J. Crawley A. P. Mikulis D. J. Davis K. D. (2001). The effect of task relevance on the cortical response to changes in visual and auditory stimuli: an event-related fMRI study. *Neuroimage* 14 1256–1267. 10.1006/nimg.2001.0946 11707082

[B20] Downar J. Crawley A. P. Mikulis D. J. Davis K. D. (2002). A cortical network sensitive to stimulus salience in a neutral behavioral context across multiple sensory modalities. *J. Neurophysiol.* 87 615–620. 10.1152/jn.00636.2001 11784775

[B21] Downar J. Mikulis D. J. Davis K. D. (2003). Neural correlates of the prolonged salience of painful stimulation. *Neuroimage* 20 1540–1551. 14642466 10.1016/s1053-8119(03)00407-5

[B22] Dworkin R. H. Turk D. C. Farrar J. T. Haythornthwaite J. A. Jensen M. P. Katz N. P. (2005). Core outcome measures for chronic pain clinical trials: IMMPACT recommendations. *Pain* 113 9–19. 10.1016/j.pain.2004.09.012 15621359

[B23] Dworkin. (1995). “Personal and societal impact of orofacial pain,” in *Orofacial Pain and Temporomandibular Disorders.* eds Fricton J. R. Dubner R. (New York: Raven Press), 15–32.

[B24] Ferdek M. A. Oosterman J. M. Adamczyk A. K. van Aken M. Woudsma K. J. Peeters B. W. M. M. (2019). Effective Connectivity of Beta Oscillations in Endometriosis-Related Chronic Pain During rest and Pain-Related Mental Imagery. *J. Pain* 113:9–19. 10.1016/j.jpain.2019.05.011 31152855

[B25] Fillingim R. B. Slade G. D. Greenspan J. D. Dubner R. Maixner W. Bair E. (2018). Long-term changes in biopsychosocial characteristics related to temporomandibular disorder: findings from the OPPERA study. *Pain* 159 2403–2413. 10.1097/j.pain.0000000000001348 30028791 PMC6193833

[B26] Fischer A. A. (1987). Pressure algometry over normal muscles. Standard values, validity and reproducibility of pressure threshold. *Pain* 30 115–126. 10.1016/0304-3959(87)90089-3 3614975

[B27] Fregni F. Boggio P. S. Lima M. C. Ferreira M. J. L. Wagner T. Rigonatti S. P. (2006). A sham-controlled, phase II trial of transcranial direct current stimulation for the treatment of central pain in traumatic spinal cord injury. *Pain* 122 197–209. 10.1016/j.pain.2006.02.023 16564618

[B28] Greenspan J. D. Slade G. D. Bair E. Dubner R. Fillingim R. B. Ohrbach R. (2011). Pain Sensitivity Risk Factors for Chronic TMD: Descriptive Data and Empirically Identified Domains from the OPPERA Case Control Study. *J. Pain* 12(Suppl. 11), T61–T74. 10.1016/j.jpain.2011.08.006 22074753 PMC3249228

[B29] Hansen T. M. Mark E. B. Olesen S. S. Gram M. Frøkjær J. B. Drewes A. M. (2017). Characterization of cortical source generators based on electroencephalography during tonic pain. *J. Pain Res.* 10 1401–1409. 10.2147/JPR.S132909 28652806 PMC5476635

[B30] Harte S. E. Harris R. E. Clauw D. J. (2018). The neurobiology of central sensitization. *J. Applied Biobehav. Res.* 23:e12137 10.1111/jabr.12137

[B31] Hauck M. Lorenz J. Engel A. K. (2007). Attention to Painful Stimulation Enhances γ-Band Activity and Synchronization in Human Sensorimotor Cortex. *J. Neurosci.* 27 9270–9277. 10.1523/JNEUROSCI.2283-07.2007 17728441 PMC6673131

[B32] Jensen M. P. Sherlin L. H. Gertz K. J. Braden A. L. Kupper A. E. Gianas A. (2013). Brain EEG activity correlates of chronic pain in persons with spinal cord injury: clinical implications. *Spinal Cord* 51 55–58. 10.1038/sc.2012.84 22801188

[B33] Kim J.-Y. Kim S.-H. Seo J. Kim S.-H. Han S. W. Nam E. J. (2013). Increased power spectral density in resting-state pain-related brain networks in fibromyalgia. *Pain* 154 1792–1797. 10.1016/j.pain.2013.05.040 23714266

[B34] Kucyi A. Davis K. D. (2015). The dynamic pain connectome. *Trends Neurosci.* 38 86–95. 10.1016/j.tins.2014.11.006 25541287

[B35] Kucyi A. Hodaie M. Davis K. D. (2012). Lateralization in intrinsic functional connectivity of the temporoparietal junction with salience- and attention-related brain networks. *J. Neurophysiol.* 108 3382–3392. 10.1152/jn.00674.2012 23019004

[B36] Lautenbacher S. Rollman G. B. McCain G. A. (1994). Multi-method assessment of experimental and clinical pain in patients with fibromyalgia. *Pain* 59 45–53. 10.1016/0304-3959(94)90046-9 7854801

[B37] Lépine J.-P. Briley M. (2004). The epidemiology of pain in depression. *Hum. Psychophar.* 19(Suppl. 1), S3–S7. 10.1002/hup.618 15378670

[B38] Lim M. Kim J. S. Kim D. J. Chung C. K. (2016). Increased Low- and High-Frequency Oscillatory Activity in the Prefrontal Cortex of Fibromyalgia Patients. *Front. Hum. Neurosci.* 10:111. 10.3389/fnhum.2016.00111 27014041 PMC4789463

[B39] Llinás R. R. Ribary U. Jeanmonod D. Kronberg E. Mitra P. P. (1999). Thalamocortical dysrhythmia: A neurological and neuropsychiatric syndrome characterized by magnetoencephalography. *Proc. Natl. Acad. Sci. U S A.* 96 15222–15227. 10.1073/pnas.96.26.15222 10611366 PMC24801

[B40] Loeser J. D. Treede R.-D. (2008). The Kyoto protocol of IASP Basic Pain Terminology. *Pain* 137 473–477. 10.1016/j.pain.2008.04.025 18583048

[B41] Mansour A. R. Farmer M. A. Baliki M. N. Apkarian A. V. (2014). Chronic pain: the role of learning and brain plasticity. *Restor. Neurol. Neurosci.* 32 129–139. 10.3233/RNN-139003 23603439 PMC4922795

[B42] Maquet D. Croisier J.-L. Demoulin C. Crielaard J.-M. (2004). Pressure pain thresholds of tender point sites in patients with fibromyalgia and in healthy controls. *Eur. J. Pain* 8 111–117. 10.1016/S1090-3801(03)00082-X 14987620

[B43] May E. S. Nickel M. M. Ta Dinh S. Tiemann L. Heitmann H. Voth I. (2019). Prefrontal gamma oscillations reflect ongoing pain intensity in chronic back pain patients. *Hum. Brain Mapp.* 40 293–305. 10.1002/hbm.24373 30260531 PMC6585682

[B44] Mouraux A. Diukova A. Lee M. C. Wise R. G. Iannetti G. D. (2011). A multisensory investigation of the functional significance of the “pain matrix.”. *Neuroimage* 54 2237–2249. 10.1016/j.neuroimage.2010.09.084 20932917

[B45] Neblett R. Cohen H. Choi Y. Hartzell M. M. Williams M. Mayer T. G. (2013). The Central Sensitization Inventory (CSI): establishing clinically significant values for identifying central sensitivity syndromes in an outpatient chronic pain sample. *J. Pain* 14 438–445. 10.1016/j.jpain.2012.11.012 23490634 PMC3644381

[B46] Neblett R. Hartzell M. M. Cohen H. Mayer T. G. Williams M. Choi Y. (2015). Ability of the central sensitization inventory to identify central sensitivity syndromes in an outpatient chronic pain sample. *Clin. J. Pain* 31 323–332. 10.1097/AJP.0000000000000113 24806467

[B47] Nie H. Graven-Nielsen T. Arendt-Nielsen L. (2009). Spatial and temporal summation of pain evoked by mechanical pressure stimulation. *Eur. J. Pain* 13 592–599. 10.1016/j.ejpain.2008.07.013 18926745

[B48] Nijs J. Torres-Cueco R. van Wilgen C. P. Girbes E. L. Struyf F. Roussel N. (2014). Applying modern pain neuroscience in clinical practice: criteria for the classification of central sensitization pain. *Pain Physician.* 17 447–457. 25247901

[B49] Peng W. Babiloni C. Mao Y. Hu Y. (2015). Subjective pain perception mediated by α rhythms. *Biol. Psychol.* 109 141–150. 10.1016/j.biopsycho.2015.05.004 26026894

[B50] Pinheiro E. S. dos S. de Queirós F. C. Montoya P. Santos C. L. do Nascimento M. A. (2016). Electroencephalographic Patterns in Chronic Pain: A Systematic Review of the Literature. *PLoS One* 11:e0149085. 10.1371/journal.pone.0149085 26914356 PMC4767709

[B51] Prichep L. S. John E. R. Howard B. Merkin H. Hiesiger E. M. (2011). Evaluation of the pain matrix using EEG source localization: a feasibility study. *Pain Med.* 12 1241–1248. 10.1111/j.1526-4637.2011.01191.x 21810167

[B52] Prichep L. S. Shah J. Merkin H. Hiesiger E. M. (2018). Exploration of the Pathophysiology of Chronic Pain Using Quantitative EEG Source Localization. *Clin. EEG Neurosci.* 49 103–113. 10.1177/1550059417736444 29108430

[B53] Quartana P. J. Campbell C. M. Edwards R. R. (2009). Pain catastrophizing: a critical review. *Expert Rev. Neurother.* 9 745–758. 10.1586/ern.09.34 19402782 PMC2696024

[B54] Rodriguez-Raecke R. Niemeier A. Ihle K. Ruether W. May A. (2013). Structural brain changes in chronic pain reflect probably neither damage nor atrophy. *PLoS One* 8:e54475. 10.1371/journal.pone.0054475 23405082 PMC3566164

[B55] Sarlani E. Greenspan J. D. (2005). Why look in the brain for answers to temporomandibular disorder pain? *Cells Tissues Organs* 180 69–75. 10.1159/000086200 16088135

[B56] Sarnthein J. Jeanmonod D. (2008). High thalamocortical theta coherence in patients with neurogenic pain. *NeuroImage* 39 1910–1917. 10.1016/j.neuroimage.2007.10.019 18060808

[B57] Sarnthein J. Stern J. Aufenberg C. Rousson V. Jeanmonod D. (2006). Increased EEG power and slowed dominant frequency in patients with neurogenic pain. *Brain* 129 55–64. 10.1093/brain/awh631 16183660

[B58] Schabrun S. M. Elgueta-Cancino E. L. Hodges P. W. (2017). Smudging of the Motor Cortex Is Related to the Severity of Low Back Pain. *Spine* 42 1172–1178. 10.1097/BRS.0000000000000938 25893342

[B59] Schabrun S. M. Hodges P. W. Vicenzino B. Jones E. Chipchase L. S. (2015). Novel adaptations in motor cortical maps: the relation to persistent elbow pain. *Med. Sci. Sports Exerc.* 47 681–690. 10.1249/MSS.0000000000000469 25102290

[B60] Schiffman E. Ohrbach R. Truelove E. Look J. Anderson G. Goulet J.-P. (2014). Diagnostic Criteria for Temporomandibular Disorders (DC/TMD) for Clinical and Research Applications: recommendations of the International RDC/TMD Consortium Network and Orofacial Pain Special Interest Group†. *J. Oral. Facial Pain Headac.* 28 6–27. 10.11607/jop.1151 24482784 PMC4478082

[B61] Schulz E. May E. S. Postorino M. Tiemann L. Nickel M. M. Witkovsky V. (2015). Prefrontal Gamma Oscillations Encode Tonic Pain in Humans. *Cereb. Cortex* 25 4407–4414. 10.1093/cercor/bhv043 25754338 PMC4816790

[B62] Seeley W. W. Menon V. Schatzberg A. F. Keller J. Glover G. H. Kenna H. (2007). Dissociable intrinsic connectivity networks for salience processing and executive control. *J. Neurosci.* 27 2349–2356. 10.1523/JNEUROSCI.5587-06.2007 17329432 PMC2680293

[B63] Slade G. D. Sanders A. E. Ohrbach R. Fillingim R. B. Dubner R. Gracely R. H. (2014). Pressure pain thresholds fluctuate with, but do not usefully predict, the clinical course of painful temporomandibular disorder. *Pain* 155 2134–2143. 10.1016/j.pain.2014.08.007 25130428 PMC4197095

[B64] Smart K. M. Blake C. Staines A. Doody C. (2012). Self-reported pain severity, quality of life, disability, anxiety and depression in patients classified with “nociceptive”, “peripheral neuropathic” and “central sensitisation” pain. The discriminant validity of mechanisms-based classifications of low back (±leg) pain. *Man. Ther.* 17 119–125. 10.1016/j.math.2011.10.002 22074733

[B65] Stahl S. Briley M. (2004). Understanding pain in depression. *Hum. Psychophar.* 19(Suppl. 1), S9–S13. 10.1002/hup.619 15378669

[B66] Stern J. Jeanmonod D. Sarnthein J. (2006). Persistent EEG overactivation in the cortical pain matrix of neurogenic pain patients. *Neuroimage* 31 721–731. 10.1016/j.neuroimage.2005.12.042 16527493

[B67] Straudi S. Buja S. Baroni A. Pavarelli C. Pranovi G. Fregni F. (2018). The effects of transcranial direct current stimulation (tDCS) combined with group exercise treatment in subjects with chronic low back pain: a pilot randomized control trial. *Clin. Rehabil.* 32 1348–1356. 10.1177/0269215518777881 29783893

[B68] Sullivan M. J. L. Bishop S. R. Pivik J. (1995). The Pain Catastrophizing Scale: Development and validation. *Psychol. Assess.* 7 524–532. 10.1037/1040-3590.7.4.524

[B69] Svensson P. Baad-Hansen L. Pigg M. List T. Eliav E. Ettlin D. (2011). Guidelines and recommendations for assessment of somatosensory function in oro-facial pain conditions–a taskforce report. *J. Oral. Rehabil.* 38 366–394. 10.1111/j.1365-2842.2010.02196.x 21241350

[B70] Te M. Baptista A. F. Chipchase L. S. Schabrun S. M. (2017). Primary Motor Cortex Organization Is Altered in Persistent Patellofemoral Pain. *Pain Med.* 18 2224–2234. 10.1093/pm/pnx036 28340134

[B71] Thompson T. Correll C. U. Gallop K. Vancampfort D. Stubbs B. (2016). Is Pain Perception Altered in People With Depression? A Systematic Review and Meta-Analysis of Experimental Pain Research. *J. Pain* 17 1257–1272. 10.1016/j.jpain.2016.08.007 27589910

[B72] Tiemann L. Schulz E. Gross J. Ploner M. (2010). Gamma oscillations as a neuronal correlate of the attentional effects of pain. *Pain* 150 302–308. 10.1016/j.pain.2010.05.014 20558000

[B73] Treede R.-D. Rief W. Barke A. Aziz Q. Bennett M. I. Benoliel R. (2015). A classification of chronic pain for ICD-11. *Pain* 156 1003–1007. 10.1097/j.pain.0000000000000160 25844555 PMC4450869

[B74] Treede R.-D. Rief W. Barke A. Aziz Q. Bennett M. I. Benoliel R. (2019). Chronic pain as a symptom or a disease: the IASP Classification of Chronic Pain for the International Classification of Diseases (ICD-11). *Pain* 160 19–27. 10.1097/j.pain.0000000000001384 30586067

[B75] Uddin L. Q. (2015). Salience processing and insular cortical function and dysfunction. *Nat. Rev. Neurosci.* 16 55–61. 10.1038/nrn3857 25406711

[B76] Uhlhaas P. J. Pipa G. Lima B. Melloni L. Neuenschwander S. Nikolić D. (2009). Neural synchrony in cortical networks: history, concept and current status. *Front. Integr. Neurosci.* 3:17. 10.3389/neuro.07.017.2009 19668703 PMC2723047

[B77] von Elm E. Altman D. G. Egger M. Pocock S. J. Gøtzsche P. C. Vandenbroucke J. P. (2014). The Strengthening the Reporting of Observational Studies in Epidemiology (STROBE) Statement: guidelines for reporting observational studies. *Int. J. Surg.* 12 1495–1499. 10.1016/j.ijsu.2014.07.013 25046131

[B78] Von Knorring L. Perris C. Eisemann M. Eriksson U. Perris H. (1983). Pain as a symptom in depressive disorders: I. Relationship to diagnostic subgroup and depressive symptomatology. *Pain* 15 19–26. 10.1016/0304-3959(83)90003-9

[B79] Von Korff M. Dworkin S. F. Le Resche L. Kruger A. (1988). An epidemiologic comparison of pain complaints. *Pain* 32 173–183. 10.1016/0304-3959(88)90066-8 3362555

[B80] Welch P. D. (1967). The use of fast Fourier transform for the estimation of power spectra: a method based on time averaging over short, modified periodograms. *IEEE Trans. Aud. Electroacoust.* 15 70–73. 10.1109/TAU.1967.1161901

[B81] Whitham E. M. Pope K. J. Fitzgibbon S. P. Lewis T. Clark C. R. Loveless S. (2007). Scalp electrical recording during paralysis: quantitative evidence that EEG frequencies above 20 Hz are contaminated by EMG. *Clin. Neurophysiol.* 118 1877–1888. 10.1016/j.clinph.2007.04.027 17574912

[B82] Woolf C. J. (2011). Central sensitization: implications for the diagnosis and treatment of pain. *Pain* 152 S2–S15. 10.1016/j.pain.2010.09.030 20961685 PMC3268359

[B83] Zhou R. Wang J. Qi W. Liu F.-Y. Yi M. Guo H. (2018). Elevated Resting State Gamma Oscillatory Activities in Electroencephalogram of Patients With Post-herpetic Neuralgia. *Front. Neurosci.* 12:750. 10.3389/fnins.2018.00750 30405337 PMC6205978

